# The uptake of the pharmacy-dispensed naloxone kit program in Ontario: A population-based study

**DOI:** 10.1371/journal.pone.0223589

**Published:** 2019-10-18

**Authors:** Beatrice Choremis, Tonya Campbell, Mina Tadrous, Diana Martins, Tony Antoniou, Tara Gomes

**Affiliations:** 1 Queen’s University, Kingston, Ontario, Canada; 2 ICES, Toronto, Ontario, Canada; 3 Leslie Dan Faculty of Pharmacy, University of Toronto, Toronto, Ontario, Canada; 4 Women’s College Hospital, Toronto, Ontario, Canada; 5 Keenan Research Centre of the Li Ka Shing Knowledge Institute, St. Michael’s Hospital, Toronto, Ontario, Canada; 6 Department of Family and Community Medicine, St. Michael’s Hospital and University of Toronto, Toronto, Ontario, Canada; 7 Institute of Health Policy, Management, and Evaluation, University of Toronto, Toronto, Ontario, Canada; Ottawa Hospital Research Institute, CANADA

## Abstract

**Background:**

Naloxone is a life-saving antidote for opioid overdoses. In June 2016, the Ontario government implemented the Ontario Naloxone Program for Pharmacies (ONPP) to enhance access to naloxone.

**Objective:**

We examined the initial uptake of naloxone through the ONPP and characteristics of the individuals receiving and pharmacies dispensing naloxone kits.

**Methods:**

We conducted a population-based study of all Ontario residents who received a naloxone kit between July 1, 2016 and March 31, 2018. This involved 1) a cross-sectional analysis of monthly rates of kits dispensed; and 2) a descriptive analysis of all individuals and pharmacies who accessed and dispensed naloxone, respectively. We stratified individuals according to their opioid exposure as: prescription opioid agonist therapy (OAT) recipients, prescription opioid recipients, those with past opioid exposure and those with no/unknown opioid exposure. We calculated a Lorenz curve comparing the cumulative percent of naloxone-dispensing pharmacies and cumulative percent of naloxone kits dispensed and the corresponding Gini coefficient.

**Results:**

Naloxone dispensing through the ONPP increased considerably from 1.9 to 54.3 kits per 100,000 residents over the study period. In this time, 2,729 community pharmacies dispensed 91,069 kits to 67,910 unique individuals. Uptake was highest among prescription OAT recipients (40.7% of OAT recipients dispensed at least one kit), compared with 1.6% of prescription opioid recipients, 1.0% of those with past opioid exposure and 0.3% with no/unknown opioid exposure. Naloxone dispensing was highly clustered among pharmacies (Gini = 0.78), with 55.6% of Ontario pharmacies dispensing naloxone, and one-third (33.7%) of kits dispensed by the top 1.0% of naloxone-dispensing pharmacies.

**Conclusion:**

The ONPP launch led to a rapid increase in the number of naloxone kits dispensed in Ontario. Although the program successfully engaged people prescribed OAT, efforts to increase uptake among others at risk of opioid overdose appear warranted. Opportunities for expanding pharmacy participation should be identified and pursued.

## Introduction

Opioid-related mortality has become a major public health concern in Canada, with approximately 4,000 opioid-related deaths occurring nationally in 2017[1] and over one-quarter of these (N = 1,265) representing Ontario residents.[2] Furthermore, the number of opioid-related deaths in Ontario has increased more than 778% since 1991, and in 2015 represented 1 in 133 deaths among Ontario residents.[3–5] Although several legislative and educational interventions have been undertaken to counter escalating rates of opioid overdose and death,[6, 7] these rates continue to grow.[2] Consequently, enhanced access to harm reduction interventions is increasingly advocated for mitigating the risk of opioid overdose and death.

Naloxone is an opioid antagonist medication that can prevent death when administered during an opioid overdose with few adverse effects beyond the induction of withdrawal symptoms.[8] Because of its efficacy and safety, increased access to take-home naloxone has been endorsed by the World Health Organization.[9] As a result, many jurisdictions and agencies within Canada, the United States and the United Kingdom have introduced take-home naloxone programs as part of their opioid overdose harm reduction strategies,[6, 8] which have been generally well-received by the public and stakeholders.[6, 10, 11] Evaluations of take-home naloxone programs have found that they reduce opioid-related mortality[8] and encourage bystanders to take action.[12] Importantly, these interventions have also been found to be cost-effective in the context of naloxone provision to people using heroin.[13]

Naloxone availability in Ontario has historically been limited to specialized clinics providing care to individuals with an opioid-use disorder, public health departments, and supervised consumption sites that target populations at high risk of an overdose. However, recent evidence suggests that approximately one-quarter of opioid-related deaths in the province involve prescription opioids only[14], suggesting a need to distribute naloxone to people both with opioid use disorder and those being prescribed opioids. To increase public accessibility of naloxone, the Ontario government implemented the Ontario Naloxone Program for Pharmacies (ONPP) in June 2016 which distributes naloxone kits free of charge to all Ontarians through any community pharmacy in Ontario.[15] The ONPP automatically authorizes all operational pharmacies in Ontario to distribute naloxone, with the requirement that dispensing pharmacists must complete an online course and must counsel the patient on naloxone administration at the time of dispensing. [16] Although the goal of the ONPP is enhanced access to take-home naloxone among individuals at risk of opioid-related overdose, the uptake of this program is unknown. Our objectives were to evaluate the initial uptake of the program and to describe the characteristics of individuals accessing naloxone and pharmacies dispensing naloxone.

## Methods

### Setting

We conducted a population-based study of all Ontario residents eligible for health insurance who were dispensed a naloxone kit between July 1, 2016 and March 31, 2018.

### Data sources

We used the Ontario Drug Benefit (ODB) claims database, which captures individual-level information on all products dispensed from community pharmacies and reimbursed by the Ontario Public Drug Programs (OPDP), to identify all naloxone kits dispensed from community pharmacies across Ontario. The ODB database has been demonstrated to be complete and of high quality, with an error rate <1%.[17] This database has previously been used to monitor uptake of other pharmacy-based harm reduction programs.[18] We identified prescriptions for opioids and benzodiazepines using the Narcotics Monitoring System (NMS), a database which captures all prescriptions for controlled substances dispensed from community pharmacies in Ontario, regardless of payer. We used the Registered Persons Database (RPDB), a registry for all individuals eligible for Ontario health insurance, to determine individuals’ demographic characteristics. To capture medical comorbidities and opioid-related harms, we used the Canadian Institute for Health Information (CIHI) Discharge Abstract Database (DAD), the Ontario Mental Health Reporting System (OHMRS), the CIHI National Ambulatory Care Reporting System (NACRS), and the Ontario Health Insurance Plan (OHIP) databases, which capture inpatient hospitalizations, mental health hospitalizations, emergency department (ED) visits, and outpatient physician claims, respectively. The use of data in this project was authorized under section 45 of Ontario’s Personal Health Information Protection Act, which does not require review by a Research Ethics Board. These datasets were linked using unique encoded identifiers and analyzed at ICES in Toronto, Ontario (https://www.ices.on.ca). These databases are routinely used to study the impact of policy changes related to prescription opioids.[19, 20]

### Naloxone uptake

We conducted a cross-sectional analysis of naloxone uptake by identifying the monthly number of kits dispensed, individuals receiving at least one kit, and pharmacies dispensing naloxone over the study period. In order to differentiate naloxone uptake among different groups at risk of an opioid overdose, we stratified individuals into one of four mutually exclusive, hierarchical pre-defined groups as follows (S1 Fig). First, we defined prescription opioid agonist therapy (OAT) recipients as those with any prescription for methadone or buprenorphine/naloxone dispensed on the naloxone claim date or in the 14 days prior. Second, we defined prescription opioid recipients as those with a non-OAT opioid prescription dispensed with a days’ supply that overlapped the naloxone claim date. Third, we identified individuals with past opioid exposure as those with a history of long-term prescription opioid use or opioid-related harm. We defined these individuals as meeting at least one of the following criteria: 1) history of opioid-use disorder using OHIP claims, inpatient hospitalizations, or emergency department visits in the 5 years prior to naloxone claim date; 2) any hospitalization or emergency department visit for opioid-related harm in the 5 years prior to naloxone claim date; 3) any prescription for a long-acting non-OAT prescription opioid in the 4 years prior to naloxone claim date, and 4) five or more prescriptions for any immediate-release opioid in the 4 years prior to naloxone claim date (see S1 Table for detailed definitions of opioid use disorder and opioid-related hospital visits). Since the NMS database does not fully capture controlled substances dispensed before July 2012,[21] we were required to limit our lookback for past opioid prescriptions to 4 years. Finally, we grouped all remaining unclassified individuals as having no/unknown opioid exposure histories. Within each month, individuals who received naloxone were stratified by their opioid exposure status, as described above, defined on their naloxone claim date. If an individual received more than one naloxone kit in a given month and their opioid exposure status changed, they were included in each related exposure group for the month.

We similarly applied the hierarchical mutually exclusive stratification method for all Ontario residents to define population denominators in each month over the study period. Here, we defined prescription OAT and prescription opioid populations as those who had a prescription dispensed within the month of interest, or a prior prescription with a days’ supply overlapping the month of interest. We defined individuals with past opioid exposure using 1) the first day in the month of interest to capture past opioid prescriptions and 2) the last day in the month of interest to capture history of opioid-use disorder and opioid-related harms. All remaining individuals were defined in the no/unknown exposure group population. In our primary analysis we reported population-adjusted monthly rates of naloxone dispensing per 100,000 population overall, and stratified by opioid exposure group.

### Characteristics of individuals dispensed naloxone

We identified all individuals who received a pharmacy dispensed naloxone kit over the study period, using the most recent naloxone claim date as the index date. Within this cohort, we identified demographic characteristics (age, sex, public drug coverage eligibility, location of residence (urban/rural, southern/northern Ontario), and neighborhood income quintile), comorbidities associated with risk of opioid overdose (alcohol-use disorder, chronic obstructive pulmonary disease (COPD), kidney disease and liver disease; see S1 Table for definitions), history of opioid-use disorder and opioid-related harm (opioid-related hospitalizations or ED visits), prescription information (opioid or benzodiazepine prescription overlapping the index date, daily opioid dose measured in milligrams of morphine or equivalent (MME) at index date, number of non-OAT opioid prescriptions dispensed in the past year, and exposure to opioids indicated for pain, OAT, or as an antitussive), and total number of naloxone kits dispensed over the entire study period. We reported patient characteristics overall, and stratified by the opioid exposure groups defined above. We used standardized differences to determine whether characteristics differed between the prescription OAT recipient group and each of the other exposure groups, with a 0.1 threshold for denoting inter-group differences.[22]

### Characteristics of naloxone-dispensing pharmacies

We identified characteristics of pharmacies that dispensed naloxone over the entire study period, including the number of kits dispensed, location in Ontario (rural/urban area) and whether the pharmacy dispensed OAT. We examined clustering of naloxone dispensing through Ontario pharmacies using Lorenz curves. We calculated the Gini coefficient and the corresponding 95% confidence interval for the Lorenz curve comparing the cumulative percent of naloxone-dispensing pharmacies and cumulative percent of naloxone kits dispensed.

## Results

### Naloxone uptake

**Fig 1:**
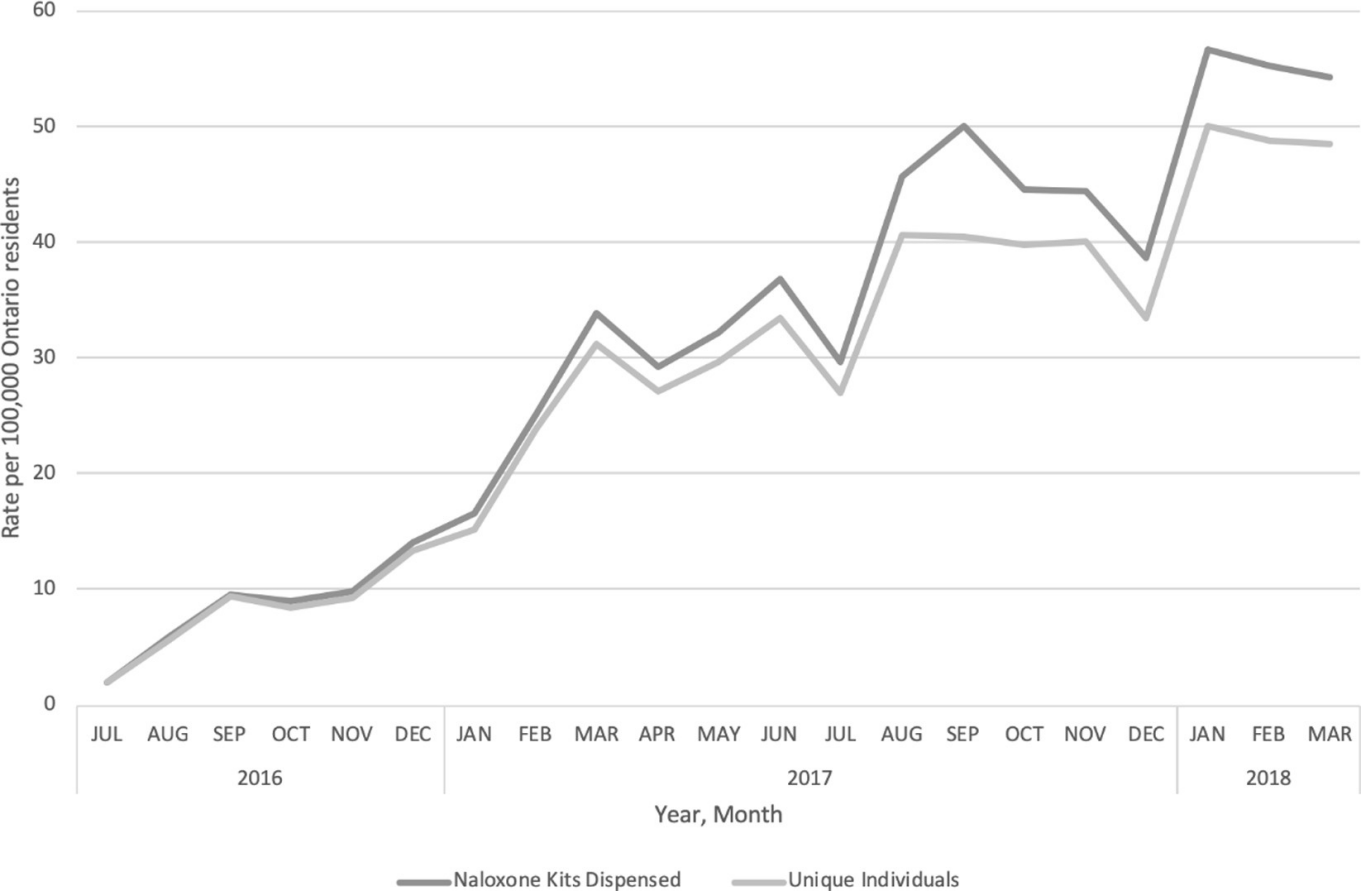
Monthly uptake of the ONPP per 100,000 Ontario residents between July 2016 and March 2018.

Between July 2016 and March 2018, 2,729 community pharmacies dispensed 91,069 naloxone kits to 67,910 unique individuals. In 2017 specifically, there were 60,375 kits dispensed to 46,610 individuals. Over the study period, the monthly rate of kits dispensed increased 29-fold from 1.9 to 54.3 naloxone kits per 100,000 population, and the rate of individuals who accessed naloxone increased 26-fold from 1.9 to 48.5 individuals per 100,000 population (**Fig 1**).

The proportion of individuals who accessed more than one kit in a single month increased from ≤2% (≤5/268; July 2016) to 8.6% (597/6,908; March 2018). Furthermore, of the 4,909 community pharmacies in Ontario the number that dispensed naloxone increased from 46 (0.9%) to 1,459 (29.7%) per month over the study period.

**Fig 2:**
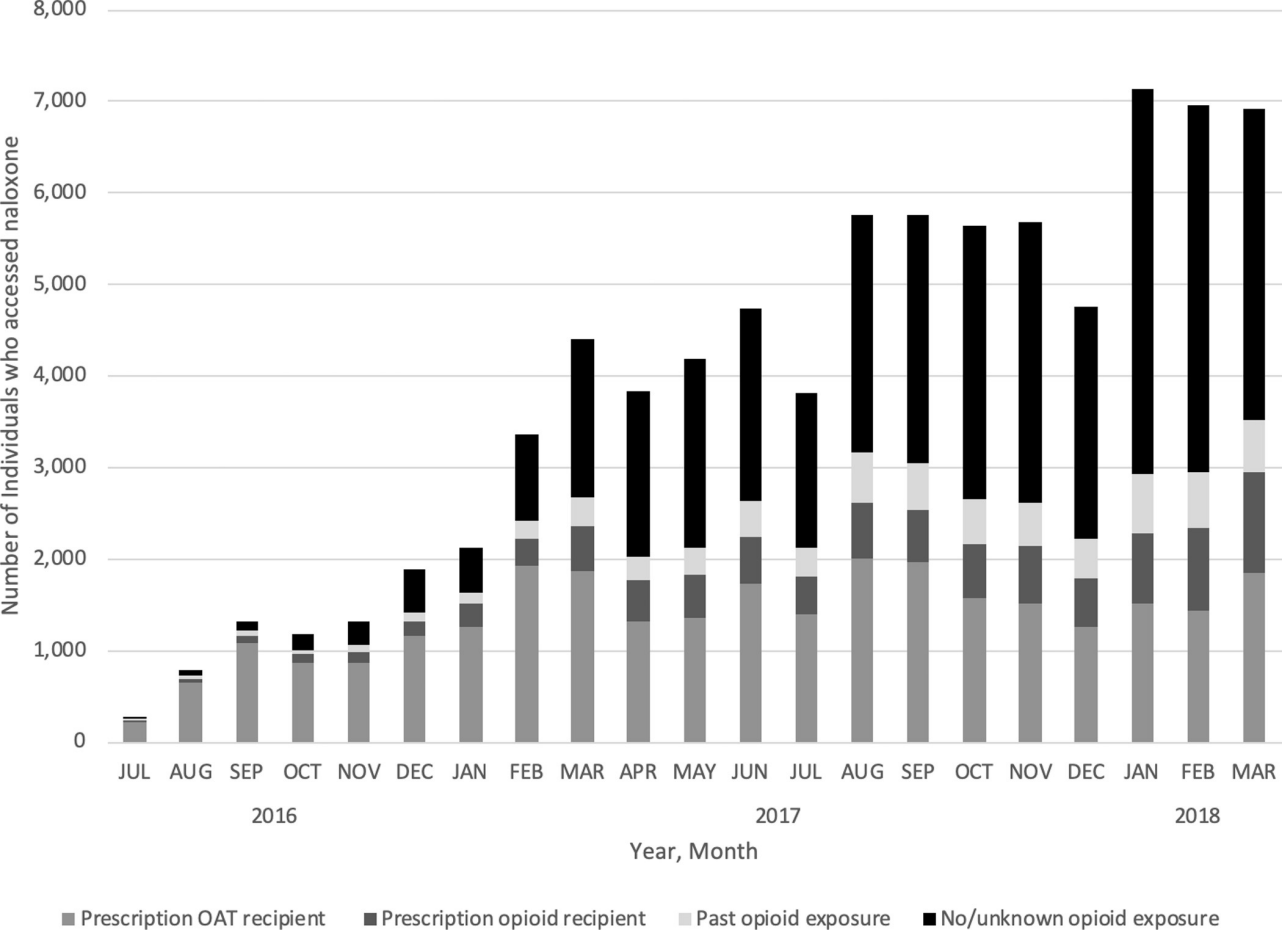
Monthly number of individuals who accessed naloxone between July 2016 and March 2018, stratified by opioid exposure group.

Over the study period, naloxone uptake was most prevalent among prescription OAT recipients, with 40.7% of all prescription OAT recipients having accessed naloxone at least once (**Table 1**). In contrast, 1.6% of prescription opioid recipients and 1.0% of those with past opioid exposure were dispensed a kit. Although only 0.3% of Ontario residents with no/unknown opioid exposure were dispensed a kit, the monthly uptake of individuals accessing naloxone in this group grew most quickly over the study period, increasing 200-fold (from 17 individuals in July 2016 to 3,399 individuals in March 2018) (**Fig 2**). Therefore, despite low population-adjusted prevalence, by March 2018, 44.0% (3,399/7,732) of all naloxone kits dispensed through the ONPP were provided to people with no/unknown opioid exposure.

**Table 1:** Characteristics of individuals who accessed naloxone between July 2016 and March 2018.

	Total	Prescription OAT recipient	Prescription opioid recipient	Past opioid exposure	No/unknown opioid exposure
Number (% of total)	67,910	19,488 (28.7%)	8,306 (12.2%)	5,297 (7.8%)	34,819 (51.3%)
**Number of naloxone kits, n (%)**					
** Mean ± SD**	1.3 ± 2.2	1.6 ± 1.8	1.1 ± 0.7*	1.5 ± 1.9*	1.2 ± 2.5*
** 1**	56,587 (83.3%)	13,616 (69.9%)	7,586 (91.3%)*	4,234 (79.9%)*	31,151 (89.5%)*
** 2–5**	10,454 (15.4%)	5,363 (27.5%)	683 (8.2%)*	950 (17.9%)*	3,458 (9.9%)*
** 6+**	869 (1.3%)	509 (2.6%)	37 (0.4%)*	113 (2.1%)	210 (0.6%)*
**Demographic characteristics**					
**Age—Median (IQR)**	38 (28–51)	37 (30–46)	53 (44–61)*	41 (31–53)*	35 (25–49)*
**Male, n (%)**	34,163 (50.3%)	11,690 (60.0%)	4,154 (50.0%)*	2,548 (48.1%)*	15,771 (45.3%)*
**OPDP eligibility, n (%)**	30,168 (44.4%)	14,792 (75.9%)	5,439 (65.5%)*	2,984 (56.3%)*	6,953 (20.0%)*
**Urban residence, n (%)**	60,517 (89.1%)	17,318 (88.9%)	7,329 (88.2%)	4,647 (87.7%)	31,223 (89.7%)
**Residence in northern Ontario, n (%)**	5,971 (8.8%)	2,612 (13.4%)	624 (7.5%)*	523 (9.9%)*	2,212 (6.4%)*
**Income quintile, n (%)**					
** 1 (lowest)**	20,165 (29.7%)	7,581 (38.9%)	2,731 (32.9%)*	1,821 (34.4%)	8,032 (23.1%)*
** 2**	13,959 (20.6%)	4,366 (22.4%)	1,786 (21.5%)	1,131 (21.4%)	6,676 (19.2%)
** 3**	11,806 (17.4%)	3,135 (16.1%)	1,524 (18.3%)	889 (16.8%)	6,258 (18.0%)
** 4**	11,012 (16.2%)	2,466 (12.7%)	1,243 (15.0%)	723 (13.6%)	6,580 (18.9%)*
** 5**	10,294 (15.2%)	1,710 (8.8%)	958 (11.5%)	654 (12.3%)*	6,972 (20.0%)*
**Comorbidities, n (%)**					
** Alcohol-use Disorder (5 years prior to index)**	7,333 (10.8%)	3,400 (17.4%)	850 (10.2%)*	1,051 (19.8%)	2,032 (5.8%)*
** COPD (any diagnosis prior to index)**	6,895 (10.2%)	2,118 (10.9%)	2,394 (28.8%)*	757 (14.3%)*	1,626 (4.7%)*
** Kidney Disease (5 years prior to index)**	1,260 (1.9%)	333 (1.7%)	475 (5.7%)*	145 (2.7%)	307 (0.9%)
** Liver Disease (5 years prior to index)**	6,979 (10.3%)	4,087 (21.0%)	1,028 (12.4%)*	790 (14.9%)*	1,074 (3.1%)*
**Opioid-related characteristics**					
**History of opioid-use disorder (5 years prior to index), n (%)**	21,885 (32.2%)	18,843 (96.7%)	994 (12.0%)*	2,048 (38.7%)*	0 (0.0%)*
**History of opioid-related** **hospitalizations or ED visits (5 years prior to index), n (%)**	2,703 (4.0%)	1,794 (9.2%)	288 (3.5%)*	621 (11.7%)	0 (0.0%)*
**Number of non-OAT opioid prescriptions (1 year prior to index), n (%)**					
** Median (IQR)**	0 (0–1)	0 (0–1)	18 (11–32)*	1 (0–3)*	0 (0–0)*
** 0**	47,696 (70.2%)	14,079 (72.2%)	270 (3.3%)*	2,555 (48.2%)*	30,792 (88.4%)*
** 1**	5,928 (8.7%)	1,748 (9.0%)	212 (2.6%)*	774 (14.6%)*	3,194 (9.2%)
** 2–5**	4,197 (6.2%)	1,527 (7.8%)	644 (7.8%)	1,193 (22.5%)*	833 (2.4%)*
** 6–10**	1,753 (2.6%)	497 (2.6%)	871 (10.5%)*	385 (7.3%)*	0 (0.0%)*
** 11+**	8,336 (12.3%)	1,637 (8.4%)	6,309 (76.0%)*	390 (7.4%)	0 (0.0%)*
**Current use of any opioid, n (%)**					
** Indicated for Pain**	9,358 (13.8%)	1,128 (5.8%)	8,230 (99.1%)*	0 (0.0%)*	0 (0.0%)*
** Indicated as Antitussive**	115 (0.2%)	12 (0.1%)	103 (1.2%)*	0 (0.0%)*	0 (0.0%)*
** Indicated for OAT**	16,535 (24.3%)	16,535 (84.8%)	0 (0.0%)*	0 (0.0%)*	0 (0.0%)*
Opioid daily dose at index (MME, IQR)**	90 (30–240)	60 (30–180)	90 (30–240)*	N/A	N/A
**Individuals with high daily dose at index (>90 MME), n (%)**	4,371 (6.4%)	453 (2.3%)	3,918 (47.2%)*	N/A	N/A
**Current prescription of benzodiazepines, n (%)**	7,047 (10.4%)	3,009 (15.4%)	2,356 (28.4%)*	640 (12.1%)*	1,042 (3.0%)*
**Current prescription of any opioid and benzodiazepine, n (%)**	4,976 (7.3%)	2,620 (13.4%)	2,356 (28.4%)*	0 (0.0%)*	0 (0.0%)*

IQR: interquartile range; MME: milligram morphine equivalent; OAT: opioid agonist therapy

* Significant difference compared to prescription OAT recipients (defined as a standardized difference >0.1)

**For opioids indicated for pain or as an antitussive only as opioids for OAT cannot be converted to MME using data available

### Characteristics of individuals dispensed naloxone

Among the entire cohort of 67,910 individuals dispensed naloxone over the study period, 51.3% had no/unknown opioid exposure (34,819 individuals), nearly one-third were prescription OAT recipients (28.7%; 19,488 individuals) and the remaining were prescription opioid recipients (12.2%; 8,306 individuals) and those with past opioid exposure (7.8%; 5,297 individuals) (**Table 1**). The majority of individuals dispensed naloxone over the study period accessed only one kit (83.3%; 56,587); however, a greater proportion of prescription OAT recipients and those with past opioid exposure accessed multiple kits (30.1% and 20.1%, respectively), compared with 8.7% and 10.5% of prescription opioid recipients and individuals with no/unknown opioid exposure, respectively (**Table 1**).

The median age of individuals who accessed naloxone was 38 years (interquartile range (IQR) 28 to 51) and approximately half (50.3%) were male. Most naloxone recipients resided in urban areas (89.1%) and in southern Ontario (91.2%), and approximately half resided in low-income neighborhoods (50.3% in the lowest two income quintiles). Characteristics of individuals differed by opioid exposure, with the most noticeable differences between prescription OAT recipients and the no/unknown exposure group. In comparison to prescription OAT recipients, individuals in the no/unknown exposure group were significantly more likely to be female (54.7% vs. 40.0%; standardized difference (SD) 0.30), live in the highest income neighborhoods (20.0% vs. 8.8%; SD 0.32) and have lower comorbidity rates, including alcohol-use disorder (5.8% vs. 17.4%; SD 0.37) and liver disease (3.1% vs. 21.0%; SD 0.57). Furthermore, prescription opioid recipients who accessed naloxone tended to be chronically receiving opioids (76.0% with ≥11 prescriptions in the past year), treated with high daily opioid doses (47.2% with >90 MME), and receiving current benzodiazepine prescriptions (28.4%; **Table 1**).

### Characteristics of naloxone-dispensing pharmacies

Over the study period, 2,729 pharmacies (55.6% of all Ontario community pharmacies) dispensed naloxone, and among these pharmacies, naloxone dispensing was highly clustered (Gini coefficient 0.78; 95% CI, 0.73–0.83); **S2 Fig**). Specifically, one-third (33.7%) of naloxone kits were dispensed by the top 1.0% (N = 35) of naloxone-dispensing pharmacies, and 88.0% of naloxone-dispensing pharmacies dispensed under 50 kits over the entire study period. The majority of naloxone-dispensing pharmacies were located in urban areas (86.9%) and dispensed OAT (66.9%) (**Table 2**).

**Table 2:** Characteristics of pharmacies dispensing naloxone between July 2016 and March 2018.

	Naloxone-dispensing pharmacies n = 2,729
% of all Ontario pharmacies (n = 4,909)	55.6%
Pharmacy characteristics	
Number of naloxone kits dispensed per pharmacy–Median (IQR)	8 (3–23)
Low (<15), n (%)	1,792 (65.7%)
Medium (15–49), n (%)	608 (22.3%)
High (50+), n (%)	329 (12.1%)
Rural pharmacy, n (%)	356 (13.1%)
OAT-dispensing pharmacy, n (%)	1,826 (66.9%)

IQR: interquartile range

## Discussion

In this population-based study, we found that 67,910 individuals were dispensed naloxone through a community pharmacy in the first 21 months of the ONPP. The program was most successful in providing access to prescription OAT recipients, with less uptake among prescription opioid recipients and individuals with past opioid exposure despite their risk of overdose. Furthermore, although the population-adjusted rate of dispensing was low among people with no/unknown opioid exposure, this group was responsible for more than half of all naloxone kits were dispensed over the study period. Importantly, we also found that naloxone dispensing was highly clustered, with just over half of Ontario pharmacies participating in the ONPP, suggesting that barriers to access may continue to exist.

The ONPP’s relative success providing naloxone access to prescription OAT recipients is important as this is a group at high risk of overdose.[23] This finding may reflect both the frequency of contact between this population and healthcare providers as well as the initial launch of the ONPP within OAT-dispensing pharmacies.[15] However, more than half of people prescribed OAT in the province were not dispensed naloxone from a pharmacy, suggesting that improvements in naloxone distribution to this high risk population may be warranted if they are not able to access naloxone from other distribution centres in the province. Although the rate of naloxone dispensing among those with no/unknown opioid exposure is low given the large size of this population, the absolute number of recipients in this exposure category is high, with 51.3% of individuals who accessed naloxone falling into this category. The composition of this group is likely heterogeneous, comprising individuals who either exclusively use non-prescribed opioids but have never experienced an overdose, or individuals who believe that they may witness an overdose (e.g. family or friends of people who use opioids), a possibility supported by the demographic characteristics and low comorbidity burden of this group. The observed growth in naloxone uptake in these populations is encouraging, as it suggests that the ONPP is providing access to individuals who may witness an opioid overdose and be in a position to administer this product.[6, 12] Future work is needed to better characterize this group, their reasons for accessing naloxone, and to compare the facilitators or barriers to naloxone access that exists for different patient populations through the ONPP.

Although we found that the ONPP led to considerable growth in naloxone distribution in the province, there were some notable gaps. Specifically, naloxone uptake among prescription opioid recipients was only 1.6%, despite some of these individuals likely being at risk of overdose.[24] Prescription opioid recipients who accessed naloxone generally had prescription opioid profiles that would put them at increased risk of experiencing an opioid overdose (76.0% had received ≥11 opioid prescriptions in the past year, 47.2% received a daily dose >90 MME and 28.4% concurrently received a benzodiazepine prescription). However, naloxone uptake within this higher-risk prescription opioid recipient population remains low. Specifically, over our 21 month study period, only 3,918 naloxone recipients were receiving high dose daily opioid prescriptions (>90 MME), despite the fact that more than 55,000 Ontarians were treated with high dose opioids in 2017.[25] This suggests that a large gap in naloxone access could exist among individuals at risk of overdose due to high dose opioid prescribing, reflecting an underappreciation of the risk of overdose within this population.

At the provider level, only half (55.6%) of pharmacies eligible to participate in the ONPP did so, with a small number of pharmacies dispensing the majority of kits. Importantly, we found that two-thirds of naloxone-dispensing pharmacies also dispensed OAT, which may be influenced by the launch of the program that prioritized OAT-dispensing pharmacies for naloxone distribution.[15] These findings align with results of a 2017 survey of Canadian pharmacies demonstrating naloxone availability in only 26.9% of Ontario pharmacies,[26] and suggests that barriers to pharmacy-dispensed naloxone continue to exist across the province. Low pharmacy participation could be related to pharmacists’ reluctance to stock or recommend naloxone due to perceived lack of demand,[26] stigma surrounding opioid-use disorder[27] or underestimation of overdose risk in prescription opioid recipients.

## Limitations

Several limitations of this study warrant discussion. First, our study did not capture the entire population uptake of naloxone in Ontario since people can access this medication from other sources, including mobile services, needle exchange and hepatitis C programs, correctional facilities, safe injection sites and public health units.[28, 29] Between July 2017 and June 2018, 62% of all naloxone in Ontario was distributed through the ONPP.[30] However, individuals who receive prescription opioids may not avail themselves of naloxone from these non-pharmacy sources, and therefore our estimates in this population are likely accurate.[31] Furthermore, this analysis provides important information regarding patterns of naloxone uptake when this product is made freely available at retail pharmacies, a model that is being considered in other jurisdictions across Canada.[16] Second, there may be some misclassification in our exposure group definitions. In particular, prior opioid use could be misclassified if it occurred before the establishment of the NMS in July 2012, or if individuals did not present an Ontario health card (2.9% of prescriptions dispensed in the NMS). Third, this study only analyzed naloxone dispensing and we do not know how many kits were used to help reverse an overdose. Therefore, an outcome-based evaluation should be conducted to assess whether the ONPP had an impact on the rate of fatal opioid overdoses.

## Conclusions

The launch of the ONPP led to a rapid increase in the number of naloxone kits dispensed from pharmacies in Ontario. Although the program successfully engaged a considerable proportion of people treated with OAT, efforts to increase uptake among individuals at risk of opioid overdose appears warranted, particularly those being prescribed high daily doses of prescription opioids. Further, opportunities to expand pharmacy participation should be identified and pursued, particularly in regions of the province experiencing high rates of opioid overdoses. As accessible health care professionals, pharmacists are ideally positioned to identify individuals at risk of opioid overdose to provide harm reduction interventions such as take-home naloxone. Future research evaluating the effectiveness of this program in reducing overdose mortality will help measure the impact of this harm reduction program.

## Supporting information

S1 Table Definition of comorbidities. *ICD-10: International Classification of Diseases, 10^th^ Revision (ICD-10) codes used to define comorbidities.
(DOCX)

S1 Fig Criteria and observation windows for opioid exposure group definitions. (DOCX)

S2 Fig Lorenz curve of naloxone kit distribution among naloxone-dispensing pharmacies between July 2016 and March 2018. Gini coefficient = 0.78.
(DOCX)
