# Supplementary material for: The uptake of the pharmacy-dispensed naloxone kit program in Ontario: A population-based study
Source: PLoS One. 2019 Oct 18;14(10):e0223589. doi: 10.1371/journal.pone.0223589 (PMC6799925; doi:10.1371/journal.pone.0223589)
Supplement: S1 Table — *ICD-10: International Classification of Diseases, 10th Revision (ICD-10) codes used to define comorbidities. (DOCX) [file pone.0223589.s001.docx]

**S1 Table. Definition of comorbidities**

| **Comorbidity** | **Database** | **Codes** |
| --- | --- | --- |
| **Alcohol-use disorder** | Emergency Department Visit (all diagnosis types), inpatient  hospitalizations (all diagnosis  types), or physician claim in the prior 5 years | Physician claims diagnosis code: 291, 303 |
|  |  | **ICD10*:** F10, K70, G31.2, G62.1, G72.1, I42.6, K29.2, K70.1, K70.4, K70.9, K86.0, Z50.2, Z71.4, Z86.40 |
| **Chronic Obstructive Pulmonary Disease** | COPD ICES-derived Cohort | Physician claims diagnosis code: 491, 492, 496  **ICD10*:** J41, J43, J44 |
| **Kidney disease** | Emergency Department Visit (all  diagnosis types), inpatient  hospitalizations (all diagnosis  types), or physician claim in the  prior 5 years | Physician claims diagnosis code - 403, 585 |
|  |  | **ICD-10*:** E102, E112, E132, E142, I12, I13, N08, N18, N19 |
| **Liver disease** | Emergency Department Visit (all diagnosis types), inpatient  hospitalizations (all diagnosis  types), or physician claim in the  prior 5 years | Physician claims diagnosis code - 070 571 573  Physician claims fee code -  Z551, Z554 |
|  |  | **ICD-10*:** B16, B17, B18, B19, I85, R17, R18, R160, R162, B942, Z225, E831, E830, K70, K713, K714, K715, K717, K721, K729, K73, K74, K753, K754, K758, K759, K76, K77 |
| **Opioid-use disorder** | Emergency Department Visit (all diagnosis types), inpatient  hospitalizations (all diagnosis  types), or physician claim in the  prior 5 years | **ICD-10:** F11 |
|  |  | **DSM-IV:** 304.00, 305.50 |
|  |  | **Feecode:** K682, K683, K684 |
| **Opioid-related hospitalization and Emergency Department visits** | Emergency Department Visit (all diagnosis types), inpatient  hospitalizations (all diagnosis  types), or physician claim in the  prior 5 years | **ICD-10*:** T400-T404 or T406 |

*ICD-10: International Classification of Diseases, 10^th^ Revision (ICD-10) codes used to define comorbidities
