# Supplementary figures and images for: The uptake of the pharmacy-dispensed naloxone kit program in Ontario: A population-based study
Source: PLoS One. 2019 Oct 18;14(10):e0223589. doi: 10.1371/journal.pone.0223589 (PMC6799925; doi:10.1371/journal.pone.0223589)

**S1 Fig. Criteria and observation windows for opioid exposure group definitions.**

**
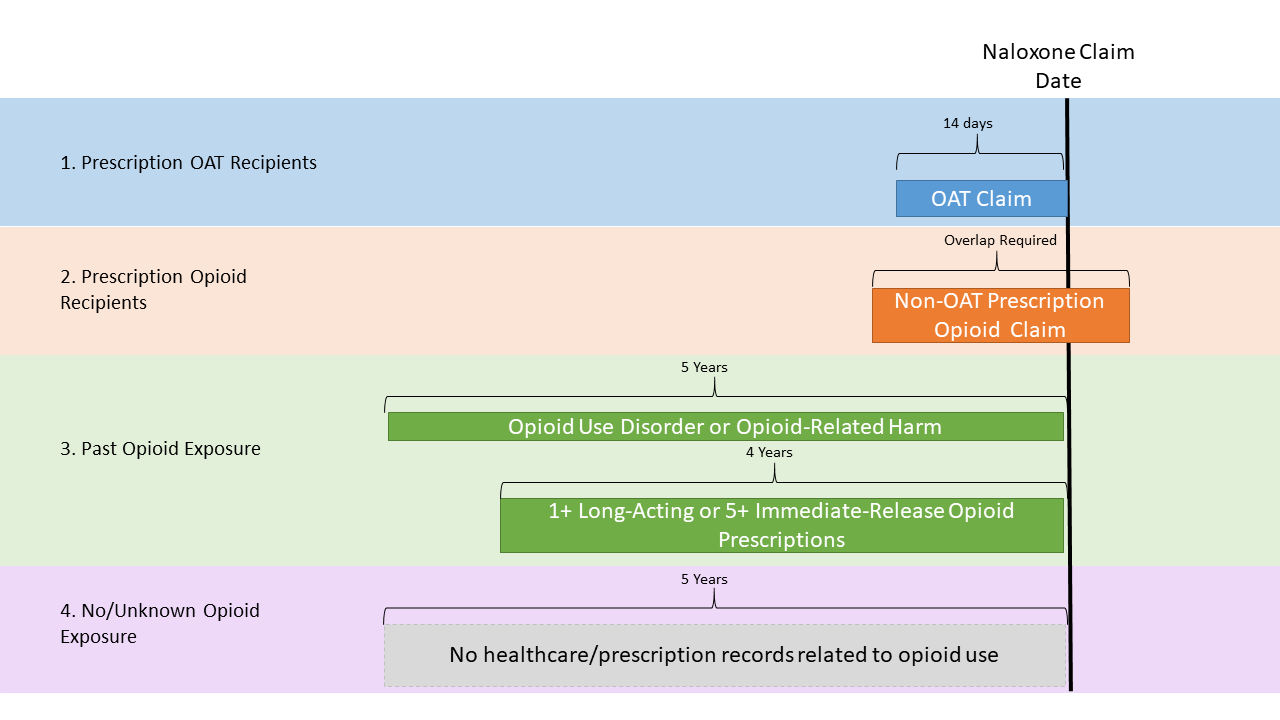
**

Supplement: S1 Fig — (DOCX) [file pone.0223589.s002.docx]
