# Supplementary material for: The uptake of the pharmacy-dispensed naloxone kit program in Ontario: A population-based study
Source: PLoS One. 2019 Oct 18;14(10):e0223589. doi: 10.1371/journal.pone.0223589 (PMC6799925; doi:10.1371/journal.pone.0223589)

**S2 Fig.** Lorenz curve of naloxone claim distribution among naloxone-dispensing pharmacies between July 2016 and March 2018 (Gini coefficient = 0.78)


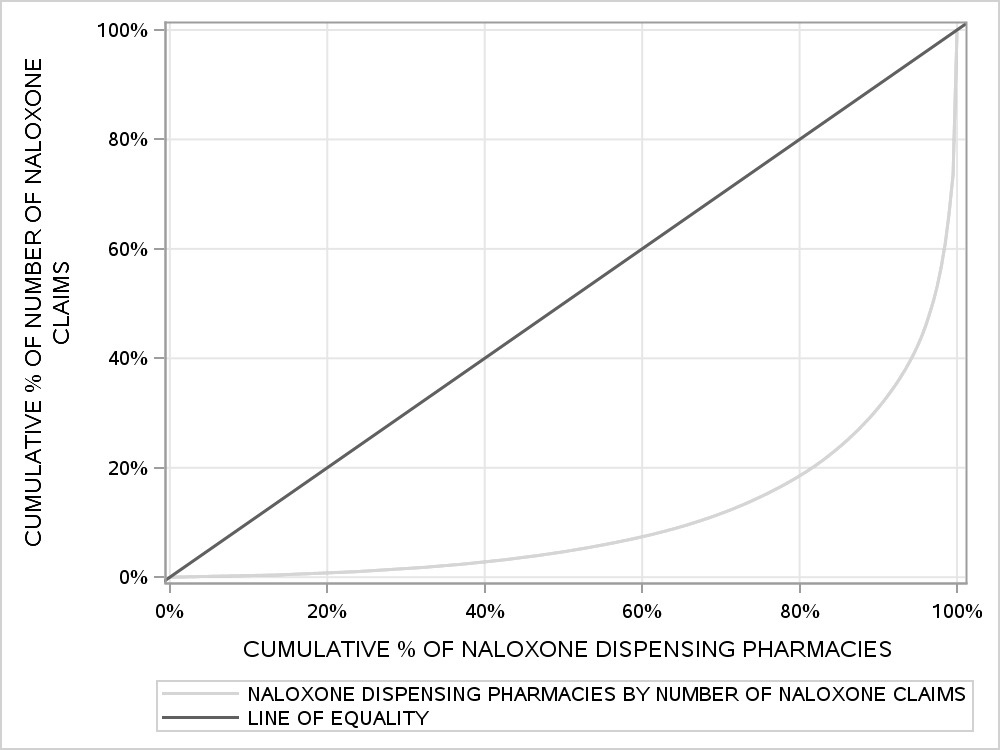

Supplement: S2 Fig — Gini coefficient = 0.78. (DOCX) [file pone.0223589.s003.docx]
